# Transcriptome and metabolome analyses of anthocyanin biosynthesis in post-harvest fruits of a full red-type kiwifruit (*Actinidia arguta*) ‘Jinhongguan’

**DOI:** 10.3389/fpls.2023.1280970

**Published:** 2023-10-09

**Authors:** Lixia Ye, Fuxi Bai, Lei Zhang, Minmin Luo, Lei Gao, Zhi Wang, Jue Peng, Qinghong Chen, Xuan Luo

**Affiliations:** ^1^Hubei Key Laboratory of Germplasm Innovation and Utilization of Fruit Trees, Institute of Fruit and Tea, Hubei Academy of Agricultural Science, Wuhan, China; ^2^College of Horticulture and Gardening, Yangtze University, Jingzhou, China

**Keywords:** *Actinidia arguta*, transcriptome, metabolome, red fruit, post-harvest, anthocyanin, cyanidin

## Abstract

Anthocyanin is the main component of pigment in red-fleshed kiwifruit. ‘Jinhongguan’ is a new cultivar of *Actinidia arguta* with red peel and flesh after harvest. However, the specific types of anthocyanin in the ‘Jinhongguan’ fruit and its biosynthesis pathways remain largely unknown. Here, the total anthocyanin content in the fruit color conversion process was determined. The results showed that total anthocyanin content increased with the deepening color of the peel and flesh. To identify the genes related to anthocyanin biosynthesis and the types of anthocyanins in the ‘Jinhongguan’ fruit, a combined analysis of transcriptome and anthocyanin-targeted metabolome was carried out. A total of 5751 common differentially expressed genes (DEGs) at different stages of peel and flesh were identified, of which 2767 were common up-DEGs and 2976 were common down-DEGs. KEGG and GO enrichment analyses showed that the common up-DEGs were significantly enriched in anthocyanin synthesis-related pathways, suggesting some up-DEGs are involved in anthocyanin biosynthesis. In total, 29 metabolites were detected in the flesh by anthocyanin-targeted metabolome. Among these, nine were differential accumulation metabolites (DAMs) in comparison to red flesh vs green flesh. Six DAMs were up-regulated, with five of them were cyanidins. The content of cyanidin-3-O-galactoside was much higher than that of other DAMs, making it the main pigment in ‘Jinhongguan’. Moreover, a total of 36 anthocyanin synthesis-related structural genes, 27 MYB transcription factors (TFs), 37 bHLH TFs and 9 WDR TFs were screened from the common DEGs. Correlation analysis of transcriptome and metabolome revealed that 9 structural genes, 6 MYB TFs, 6 bHLH TFs and 1 WDR TF were significantly associated with cyanidin-3-O-galactoside. Further, qRT-PCR analysis demonstrated that structural genes (*AaPAL3*, *Aa4CL3*, *AaCHS2*/*3*/*8*/*9*/*11*, *AaDFR1*/*2*, *AaANR1*, *UFGT3a* and *UFGT6b*) and TFs (*MYB108*, *bHLH30*, *bHLH94-1* and *WD43*) play important roles in cyanidin biosynthesis. Overall, this study identified cyanidin-3-O-galactoside as the main anthocyanin type and revealed key candidate genes of red coloration of post-harvest fruit in *Actinidia arguta*. These findings provided new insights into the color formation mechanism of post-harvest fruit and offered a theoretical basis for color regulation in kiwifruit.

## 1 Introduction

Kiwifruit belongs to Actinidia, and it has a cultivation history of more than 110 years from wild to artificial domestication (Huang and Ferguson, 2007). At present, the main cultivated species in production include *Actinidia chinensis*, *Actinidia deliciosa*, *Actinidia arguta* and *Actinidia eriantha* (Hanley, 2018; Huang et al., 2013). Kiwifruit is highly favored by consumers due to its unique fruit flavor, high nutritional value, and notably high vitamin C content (Richardson et al., 2018). The fruit of kiwifruit has a variety of colors, mainly divided into green, yellow and red, among which red fruit is mainly due to the presence of anthocyanin (Sui et al., 2013). Anthocyanins possess antioxidant capacity and exhibit multiple health-promoting advantages, such as anti-aging, anti-cancer, and lowering of blood lipids (Bendokas et al., 2020). In *Actinidia arguta* kiwifruit, the accumulation of anthocyanin is most abundant, and some varieties of them capable of accumulating anthocyanin in both the peel and flesh, resulting in the whole fruit being red or even purple, which is highly appealing to consumers (Li et al., 2018a). The molecular mechanisms of kiwifruit fruit color conversion from green to red are highly complex (Valenta et al., 2018). Currently, some genes involved in the coloration of red kiwifruit have been identified, but they mainly play a role in the pre-harvest coloring process of kiwifruit flesh (Wang et al., 2022b; Wang et al., 2022c; Shu et al., 2023). The molecular mechanisms of post-harvest reddening of kiwifruit peel and flesh remain largely unclear.

Anthocyanins widely exist in various organs of plants, such as leaves, flowers and fruits, and they are the basis for plants to present various colors. The basic structure of anthocyanin is C6-C3-C6, which is composed of two aromatic rings and one oxygen-containing heterocyclic ring (Bloor and Abrahams, 2002). According to the different structures, anthocyanin can be divided into six categories, namely, peonidin, pelargonidin, cyanidin, delphinidin, petunidin and malvidin (Smeriglio et al., 2016). Currently, about 92% of the over 500 anthocyanins are formed by combining the hydroxyl groups of these six anthocyanins with different sugar groups (Sun et al., 2018). Different types of anthocyanins present slight differences in colors. For example, pelargonidin and its derivatives are red or orange; cyanidin and its derivatives are mainly brick red or mauve; delphinidin and its derivatives are blue or purple (Liu et al., 2018). The anthocyanin biosynthesis pathway is relatively conserved in higher plants and is an important branch of flavonoid metabolism. The biosynthesis of anthocyanins can be divided into three stages according to the enzyme reaction process. The first stage is the synthesis process of 4-acyl-CoA from phenylalanine through three enzymatic reactions, which is a common pathway of secondary metabolism in many plants; the second stage is the formation of the basic skeleton of anthocyanins, and the synthesis of dihydrokaempferol by three enzymatic reactions of 4-coumaroyl A; the third stage involves the anthocyanin precursor forms different substrates under the catalysis of different enzymes, followed by gradual modification into various anthocyanin glycosides (Jaakola, 2013).

The synthesis of anthocyanins is inseparable from the catalysis of structural gene-encoded enzymes. According to the time of participation in the reaction, structural genes can be categorized into early biosynthetic genes (EBG): *phenylalanine ammonia-lyase* (*PAL*), *cinnamate 4-hydroxy-lase* (*C4H*), *4-coumaroyl-CoA* (*4CL*), *chalcone synthase* (*CHS*), *chalcone isomerase* (*CHI*), *flavanone 3-hydroxylase* (*F3H*), *flavonoid 3’-hydroxylase* (*F3’H*), *flavonoid 3’, 5’-hydroxylase* (*F3’,5’H*); and late biosynthetic genes (LBG): *dihydro flavonol 4-reductase* (*DFR*), *anthocyanidin synthase* (*ANS*), *flavonoid 3-O-glucosyl-transferase* (*UFGT*), *glutathione S-transferase* (*GST*) and so on (Liu et al., 2018). The function of these structural genes has been confirmed in various species. For instance, the expression down-regulation of *SICHS* resulted in the flavonoid content decrease and the color of the tomato peel lighter (Heredia et al., 2015). The expression up-regulation of the *DFR* gene in purple radish led to increased synthesis of anthocyanin in flavonoid metabolism, while the mutation of the *DFR* gene in green radish caused an obstacle to anthocyanin synthesis (Zhuang et al., 2019). In onion, two *ANS* allele variations resulted in the loss of anthocyanin (Kim et al., 2016).

In addition, anthocyanin synthesis is regulated by various TFs, among which the most important three types are MYB, bHLH and WDR (MBW). These TFs either individually or in combination with each other form the MBW complex, thus promoting or inhibiting anthocyanin synthesis by recognizing and binding to the promoters of structural genes (Espley et al., 2007; An et al., 2012; Zhu et al., 2015; Lai et al., 2016). For example, *MdMYBA* and *MdMYB1* regulate anthocyanin synthesis in the peel, while *MdMYB10* and *MdMYB110a* control anthocyanin synthesis in leaves and flesh (Lin-Wang et al., 2010; Hu et al., 2021). Similarly, in diploid strawberry, *FveMYB10* and *FveMYB10L* independently regulate anthocyanin synthesis in fruit and petiole, respectively, and produce different anthocyanin components (Luo et al., 2023). Both *MdbbHLH3* and *MdbbHLH33* interact with *MdbMYB10* in apple to promote fruit reddening (Espley et al., 2007). In litchi, *LcbHLH1* and *LcbHLH3* cooperate with *LcMYB1* to regulate the synthesis and accumulation of anthocyanin (Lai et al., 2016). Overexpression of the *WD40* transcription factor *StAN11* in potato can enhance the expression of the *DFR* gene and deepen the color of the tuber epidermis (Zhang et al., 2017). In pepper, silencing *CaMYBA* and *CaWD40* resulted in decreased expression of anthocyanin synthesis-related structural genes and anthocyanin content (Aguilar-Barragán and Ochoa-Alejo, 2014). In Arabidopsis, MYB family members *PAP1* and *PAP2* bind to bHLH family member *TT8* and WD40 family member *TTG1* to form a complex that activates the expression of *CHS* and *DFR* (Borevitz et al., 2000). In addition, some other TFs such as WRKY, ERF, SPL, COP1 and NAC have also been found to regulate anthocyanin synthesis (Zhou et al., 2015; Chen et al., 2019; Shu et al., 2023; Zou et al., 2023).

In kiwifruit, *AcMYB75* has been found to bind to *ANS* promoting the significant accumulation of anthocyanin in the leaves of *Actinidia chinensis* (Li et al., 2017b). *MYB110a*, which was significantly expressed in the red petal offspring of the kiwifruit hybrid population but not in the white petal individuals, could directly activate the expression of *F3GT1* (Fraser et al., 2013). Overexpression of *MYB10* or *MYB110* increased cyanidin and delphinidin anthocyanins (Peng et al., 2019; Shu et al., 2023). *MYB110* has a stronger activation effect than *MYB10*, and overexpression of *MYB10* lea to anthocyanin accumulation only in the endocarp (Wang et al., 2022a). *AcMYB5-1*/*5-2*/*A1-1* promoted the accumulation of anthocyanin in kiwifruit flesh by activating the transcription of *ANS* and *DFR* (Li et al., 2017a). While the interaction between *AcMYB123* and *AcbHLH42* is necessary for activating the expression of anthocyanin biosynthetic enzymes *AcANS* and *AcF3GT1* (Wang et al., 2019). So far, most of the genes involved in anthocyanin synthesis identified in kiwifruit are MYB TFs. The types of anthocyanins in red-fleshed kiwifruit and more key genes related to anthocyanin biosynthesis still need to be further identified.

In this study, *Actinidia arguta* ‘Jinhongguan’ is a post-harvest full-red (peel and flesh) kiwifruit cultivar, which is a good material for exploring the mechanism of anthocyanin biosynthesis in post-harvest kiwifruit. Transcriptome and metabolome were used to analyze the types and biosynthesis pathways of anthocyanins in ‘Jinhongguan’. Further, the correlation analysis between transcriptome, metabolome and qRT-PCR analysis was used to screen key candidate genes responsible for cyanidin-3-O-galactoside biosynthesis in ‘Jinhongguan’. This study identified the main types of anthocyanins in the post-harvest fruit of *Actinidia arguta* and the key candidate genes related to anthocyanin biosynthesis, which provide insight into the regulatory network of anthocyanin biosynthesis.

## 2 Materials and methods

### 2.1 Plant materials and sampling

The *Actinidia arguta* cultivar ‘Jinhongguan’ plants were grafted onto 5-year-old ‘RZ002’ (*Actinidia arguta*) rootstock and planted for more than 3 years in the National Kiwifruit Germplasm Resource Garden of China. The garden is located in the Research Institute of Fruit and Tea, Hubei Academy of Agricultural Sciences (30°29′ N, 114°16′ E), Wuhan, China. Mature fruit samples were collected in early August, with three biological duplicates, and stored in the dark at 25°C after harvest. The samples were divided into four stages based on the color of the fruit peel and flesh. Stage 1: newly harvested fruits with green peel (FP1) and flesh (FF1); Stage 2: two days after fruit harvest, the peel (FP2) began to turn red, while the flesh (FF2) was still green; Stage 3: three days after fruit harvest, the peel (FP3) became all red, and the flesh (FF3) began to turn red; Stage 4: six days after fruit harvest, both the peel (FP4) and flesh (FF4) were completely red. For the transcriptome and metabolome analyses, the fruit peel and flesh from the four stages of ‘Jinhongguan’ were separated, immediately frozen in liquid nitrogen, and then stayed at −80°C.

### 2.2 Total anthocyanin content determination

The microplate anthocyanin content determination kit (Grace Biotechnology, Suzhou, China) was used to measure the total anthocyanin content following the provided instructions. Briefly, the flesh samples were ground into a powder in liquid nitrogen, and then approximately 0.15 g sample powder was added to 1 mL extraction solution for oscillation extraction at 75°C for 25 minutes. The extract was centrifuged at 12,000 rpm for 10 minutes, and the supernatant was taken into a clean tube for testing. Take 100 μL of the supernatant and add 300 μL of reagent 1 or reagent 2 mix evenly, and then stand for 60 minutes at room temperature and in the dark. Afterward, 200 μL of the clear mixed liquid was transferred into a 96-well plate, and the absorbance at 530 nm and 700 nm was measured to obtain the A1_530_ and A1_700_ values for the mixture added with reagent 1, and the A2_530_ and A2_700_ values for the mixture added with reagent 2, respectively. The anthocyanin content was calculated using the equation: An (mg/kg) = [ΔA ÷ (ε × d) × V2 × 103 × Mr] ÷ (W × V1 ÷ V) × D × 1000 = 133.6 × ΔA ÷ W × D, where ΔA = (A1_530_ − A1_700_) − (A2_530_ − A2_700_),ε= 26900 L/mol/cm (extinction coefficient of cyanidin-3-glucoside); d = 0.5 cm (optical path), V2 = 4 × 10^-4^ L (total volume detected), Mr = 449.2 (molecular weight of cyanidin-3-glucoside), W is the sample weight, V1 = 0.1 mL (detected sample volume), V = 1 mL (sample extract volume), and D = 1 (dilution multiple). Each sample contained three biological replicates, and three measurements were taken for each biological replicate.

### 2.3 Transcriptome sequencing and analysis

A total of 12 samples were collected from three biological replicates in each stage of FP1 to FP4. Similarly, 12 samples were collected from three biological replicates in each stage of FF1 to FF4. These 24 samples from the peel and flesh of ‘Jinhongguan’ were used for transcriptome sequencing. According to the standard operating procedure, total RNA extraction was carried out using the RNAprep Plant Extraction Kit (QIAGEN, Germany), and the total amount and integrity were evaluated using the RNA Nano 6000 detection reagent system. Random hexamer primers and M-MuLV reverse transcriptase were used to synthesize the first strand cDNA, and DNA polymerase I and dNTP were used to synthesize the second strand cDNA. The DNA fragments were adenylated at the 3’end and then connected with adapters containing hairpin structures for hybridization. The library fragments were purified and optimized to obtain cDNA fragments of 370-420 bp. Then the fragments were used as templates for PCR amplification, and the amplified products were purified with AMPure XP beads. Finally, the libraries were constructed and subjected to quality control to obtain qualified libraries. The detailed steps were performed as previously study described (Pan et al., 2023).

The qualified libraries were mixed according to effective concentration and target amount and then sequenced by Illumina NovaSeq 6000. The raw reads in fastq format were first filtered by an in-house Perl script to obtain clean reads. The Q20 value, Q30 value, and GC content were calculated to evaluate the quality of the clean reads, and then the clean reads were assembled in Trinity software (v2.6.6) based on the reference sequence. Gene functional annotation was performed using the following databases: NR, NT, PFAM, KOG/COG, Swiss-Prot, KO and GO (Yu et al., 2020). Differential expression analysis was conducted using the DESeq2 R package (1.20.0) with screening thresholds of |log_2_FC| ≥1.0 and padj < 0.05. GOseq (Young et al., 2010) and KOBAS (Bu et al., 2021) were used for GO function and KEGG pathway enrichment analysis of the DEGs. Plant transcription factors were predicted by iTAK software and identified by hmmscan based on TF family classification and definition in the database. The transcriptome data was submitted to NCBI-SRA and can be accessed through NCBI BioProject with the identifier PRJNA1018137 (https://www.ncbi.nlm.nih.gov/bioproject/PRJNA1018137).

### 2.4 Anthocyanin-targeted metabolome analysis

The flesh samples were vacuum freeze-dried and ground into powder. About 50 mg of the powder was dissolved in 500 μL of extract (methanol: water: hydrochloric = 500:500:1). The mixture was vortexed for 5 minutes, followed by sonication for 5 minutes, and then centrifuged for 3 minutes (12,000 r/min, 4°C). The supernatant was collected, and the above residue was re-extracted, and then the supernatant was combined twice. The supernatant was filtered with a microporous membrane (0.22 um pore size) and the filtrate was stored in an injection vial for LC-MS/MS analysis. Ultra-high performance liquid chromatography (UPLC) (ExionLCTM AD) and tandem mass spectrometry (MS/MS) (QTRAPR6500+) were used for data collection. The specific liquid phase and mass spectrometry conditions were conducted following the methods previously reported (Shao et al., 2022).

Anthocyanins were analyzed using the Scheduled Multiple Reaction Monitoring (MRM) technique and performed by Metware Biotechnology (Wuhan, China). Analyst 1.6.3 software (Sciex) was used for data collection. All metabolite quantification was performed in Multiquant 3.0.3 software (Sciex). Anthocyanin content was detected by NetWare (http://www.metware.cn/). DAMs between groups were identified by VIP≥ 0 and |log_2_fold change| ≥ 1.0. The metabolome data has also been uploaded to the public database MetaboLights and can be accessed through the identifier MTBLS8619 (https://www.ebi.ac.uk/metabolights/MTBLS8619).

### 2.5 Metabolomic and transcriptomic integrative analysis

To jointly analyze transcriptome and metabolome data, Pearson’s Correlation Coefficients (PCC) among the DEGs and DAMs were analyzed. The PCC values were calculated using the weighted gene coexpression network analysis (WGCNA) R package with default parameters (v1.4.1717). Strong correlation conditions are |PCC| ≥ 0.8 and p < 0.05. The Cytoscape software (version 3.10.0) was used to visualize the corresponding association network. The names of the 109 anthocyanins synthesis-related structural genes and TFs in **Table S6** were mainly obtained from the annotation results of the NT and NR databases, such as *MYB4*, *MYB7* and *MYB13*. For genes that were not annotated with specific names in the NT or NR database, they were classified based on gene category and named sequentially according to the gene ID, such as *AaMYB1* to *AaMYB10*.

### 2.6 Quantitative real-time PCR analysis

The total RNA of the fruit peel and flesh samples were extracted using the Total RNA kit (Aidlab Biotechnology, Beijing, China). The quality and concentration of the RNA were detected by agarose gel electrophoresis and Nanodrop spectrophotometer. The cDNA was synthesized by TRUEscript RT MasterMix (OneStep gDNA Removal) kit (Aidlab Biotechnology, Beijing, China). The qRT-PCR was performed according to the method described in our previous report (Ye et al., 2022). The primers used in this study were listed in **Table S8**.

### 2.7 Statistical analysis

The statistical analysis was conducted in IBM SPSS Statistics software (v26.0). One-way ANOVA and Duncan test (p < 0.05) were used to determine the significant difference. The total anthocyanin content and the gene expression level were visualized in GraphPad Prism 9 software.

## 3 Results

### 3.1 Fruit peel and flesh of ‘Jinhongguan’ turn red after harvest

**Figure 1:**
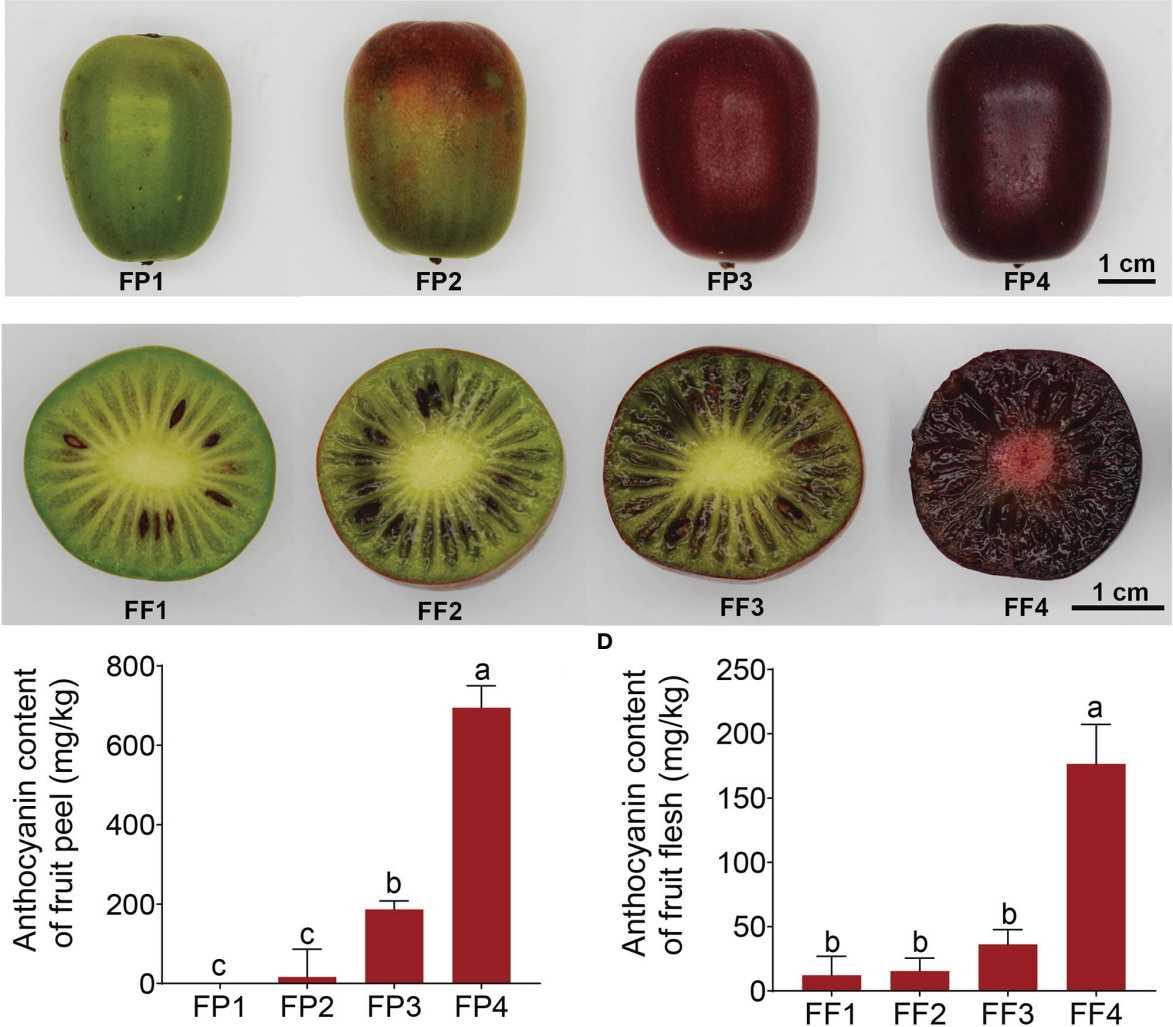
Coloration and anthocyanin content of the fruit peel and flesh in ‘Jinhongguan’. **(A, B)** The fruit peel **(A)** and flesh **(B)** color in 4 different stages after harvest. FP1-4: Stage 1 to 4 of fruit peel, FF1-4: Stage 1 to 4 in fruit flesh. **(C, D)** Total anthocyanin contents in fruit peel **(C)** and flesh **(D)** at different stages. Values represent the means ± SD from three biological replicates. The significantly different in total anthocyanin contents in fruit peel and flesh among different stages were evaluated by One-way ANOVA and Duncan test (p < 0.05). Bars with the same lowercase letters indicate no significant difference, while bars without shared lowercase letters indicate significant difference.

*‘*Jinhongguan*’* is a full-red Actinidia arguta cultivar with oval fruit, which ripens in early August in Wuhan, China. The average single fruit weight was 16.34* g*, and the average soluble solids content was 11.2%. The most typical feature of this cultivar is that the freshly mature fruit is green, and it gradually becomes full-red after being harvested and stored at 25*°*C in the dark for about one week. To explore the mechanism of post-harvest fruit color conversion, we divided the color conversion process into four stages (**Figures 1A, B**). The peel began to turn red in FP2 and became completely red in FP3 (**Figure 1A**), while the flesh began to turn red in FF3 and became entirely red in FF4 (**Figure 1B**), indicating that the peel started turning red earlier than the flesh.

It is reported that the pigments in red fruits are mainly composed of anthocyanins (Sui et al., 2013). Considering this, the total anthocyanin content in the *‘*Jinhongguan*’* fruit in four stages was measured. The results showed that the anthocyanin content was increased with increasingly deepening color (**Figures 1C, D**). The relative content of anthocyanin in FP4 and FF4 reached 694.76 mg/kg and 176.53 mg/kg fresh weight, respectively, which was significantly higher than that in FP1 and FF1 (**Figures 1C, D**). These findings suggested that the major pigment component in stage 4 of the *‘*Jinhongguan*’* fruit peel and flesh is anthocyanin, but the specific type of anthocyanin needs to be further identified.

### 3.2 Transcriptome analysis of gene expressions in fruit peel and flesh color conversion process

**Figure 2:**
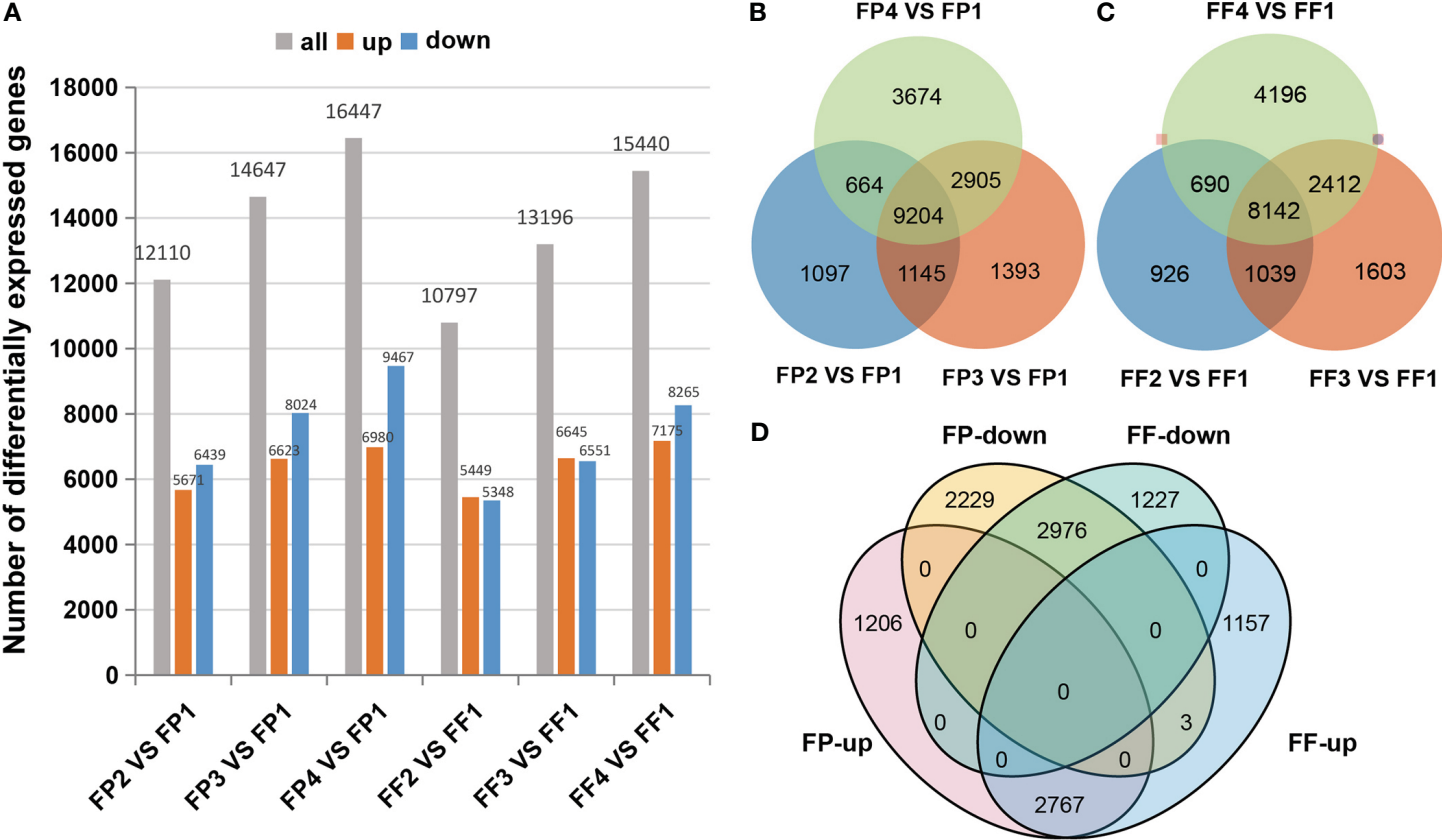
Differentially expressed genes in the transcriptome. **(A)** DEGs statistics of different stages. **(B, C)** Venn diagrams for DEGs between different stages of fruit peel **(B)** and flesh **(C)**. **(D)** Venn diagrams between co-upregulated DEGs and co-downregulated DEGs of fruit peel and flesh.

To explore the molecular mechanism of anthocyanin biosynthesis in ‘Jinhongguan’, high-throughput RNA-Seq analysis of 24 cDNA libraries of fruit peel and flesh was performed. The clean reads ranged from 19980145 to 23307997. The Q30 value of each library was more than 94%, and the average GC content was 47.10% (**Table S1**). A total of 82525 genes were identified by gene annotation in NR, NT, PFAM, KOG/COG, Swiss-Prot, KO, GO and other databases (**Table S2**). Based on the thresholds of |log_2_FC| ≥1.0 and padj < 0.05, a total of 24339 DEGs were screened with FP1 and FF1 as controls (**Table S3**). The distribution of DEGs in each stage was shown in **Figure 2**. With the red deepening of the peel and flesh, the number of identified DEGs gradually increased, and it was up to 16447 DEGs in FP4 vs FP1. Moreover, the number of DEGs in the peel was more than that in the flesh within the same stage (**Figure 2A**). Totally, 9204 DEGs were shared among the four stages of peel, and 8142 DEGs were shared among the four stages of flesh (**Figures 2B, C**). In addition, 5751 common DEGs were shared by both the peel and flesh among the four stages, including 2767 common up-DEGs and 2976 common down-DEGs (**Figure 2D**; **Table S4**). The remaining 8 common DEGs did not belong to common up-DEGs or common down-DEGs due to the inconsistent expression patterns in peel and flesh. For example, six down-DEGs in FP2 vs FP1 were up-DEGs in FF2 vs FF1 (**Figure 2D**; **Table S4**).

### 3.3 Expression pattern and function analysis of differentially expressed genes

**Figure 3:**
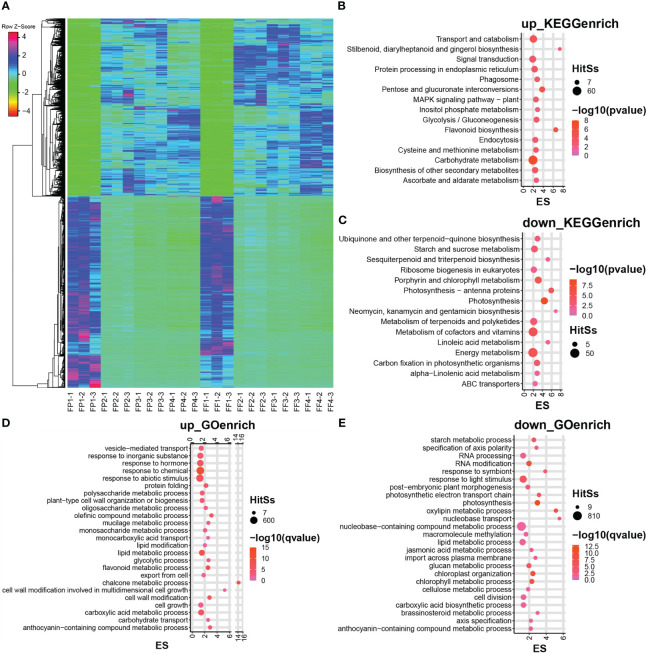
Heatmap and enrichment analysis of differentially expressed genes in the transcriptome. **(A)** Heatmap of DEGs in stage 1 to 4 of fruit peel and flesh. **(B, C)** The KEGG enrichment analysis of co-upregulated DEGs **(B)** and co-downregulated DEGs **(C)** of fruit peel and flesh. **(D, E)** The GO enrichment analysis of co-upregulated DEGs **(D)** and co-downregulated DEGs **(E)** of fruit peel and flesh.

Further, the expression levels of 5751 common DEGs in peel and flesh were analyzed. The heatmap showed that these common DEGs exhibited very consistent expression patterns between peel and flesh, as well as between different stages (**Figure 3A**). For example, a certain up-DEG tended to be up-regulated in all stages from stage 2 to stage 4, and vice versa, a down-DEG exhibited down-regulated expression in all stages (**Figure 3A**). This indicates that these DEGs were strongly positively or negatively correlated with the peel and flesh color conversion process. To further explore the function of these DEGs, KEGG and GO enrichment analyses of the common up-DEGs and common down-DEGs were performed. The KEGG enrichment analysis revealed that the common up-DEGs were mainly enriched in pathways such as carbohydrate metabolism, transport and catabolism, and signal transduction, while the common down-DEGs were mainly enriched in energy metabolism and cofactors and vitamins metabolism pathways (**Figures 3B, C**). Notably, some common up-DEGs were significantly enriched in flavonoid biosynthesis (**Figure 3B**). In terms of GO enrichment, our focus was mainly on the biological process pathways, and the results showed that the common up-DEGs were mainly enriched in response to chemical, abiotic stimulus and hormone pathways. Furthermore, these up-DEGs were also significantly enriched in flavonoid metabolic process, chalcone metabolic process and anthocyanin-containing compound metabolic process, all of which are relevant to anthocyanin biosynthesis (**Figure 3D**). On the other hand, the common down-DEGs were mainly enriched in the nucleobase-containing compound metabolic process, response to light stimulus and lipid metabolic process pathways (**Figure 3E**). It is worth noting that some common up-DEGs were significantly enriched in anthocyanin biosynthesis-related pathways in both KEGG and GO enrichment analysis (**Figures 3B, D**), and these genes might be directly involved in anthocyanin biosynthesis in the peel and flesh of ‘Jinhongguan’.

### 3.4 Anthocyanin-targeted metabolome analysis in flesh color conversion process

**Figure 4:**
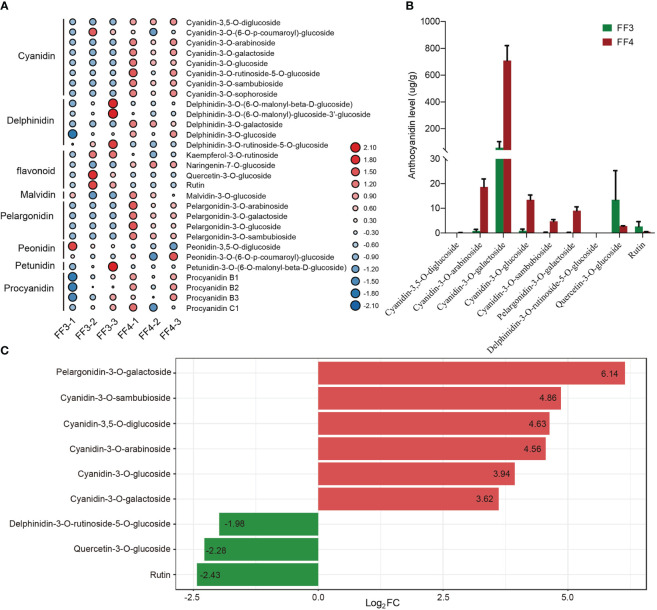
Statistical analysis of metabolome. **(A)** Heatmap of 29 anthocyanin metabolites in 8 categories identified in FF3 and FF4. **(B)** The content levels of 9 DAMs in FF3 and FF4. **(C)** Difference multiples of anthocyanins in FF3 and FF4. Red and green represent up-DAMs and down-DAMs, respectively.

To investigate the types of anthocyanin formed during the post-harvest color conversion of ‘Jinhongguan’ fruit, anthocyanin-targeted metabolome analysis was performed on stage 3 (FF3) and stage 4 (FF4) of the flesh (**Figure 1B**). The anthocyanin types in ‘Jinhongguan’ fruits were identified with 108 different anthocyanins as standard substances. A total of 29 metabolites of anthocyanin belonging to eight categories were identified, and among these categories, cyanidin was the most abundant (**Figure 4A**; **Table S5**). In the comparison between FF3 and FF4, nine DAMs were identified, of which six DAMs were up-regulated in FF4, with five being cyanidin and one being pelargonidin. Three DAMs were down-regulated, including two flavonoids and one delphinidin (**Figure 4B**). The difference multiple changes value log_2_FC of the six up-DAMs were all more than three, which was greater than that of the three down-DAMs (**Figure 4C**). Further, we investigated the content of the nine DAMs and found that the accumulation of cyanidin-3-O-galactoside in FF4 was significantly higher than that of other anthocyanins (**Figure 4B**), suggesting that cyanidin-3-O-galactoside might be the main pigment responsible for the flesh red coloration in ‘Jinhongguan’.

### 3.5 Analysis of differentially expressed structural genes involved in cyanidin biosynthesis pathway

**Figure 5:**
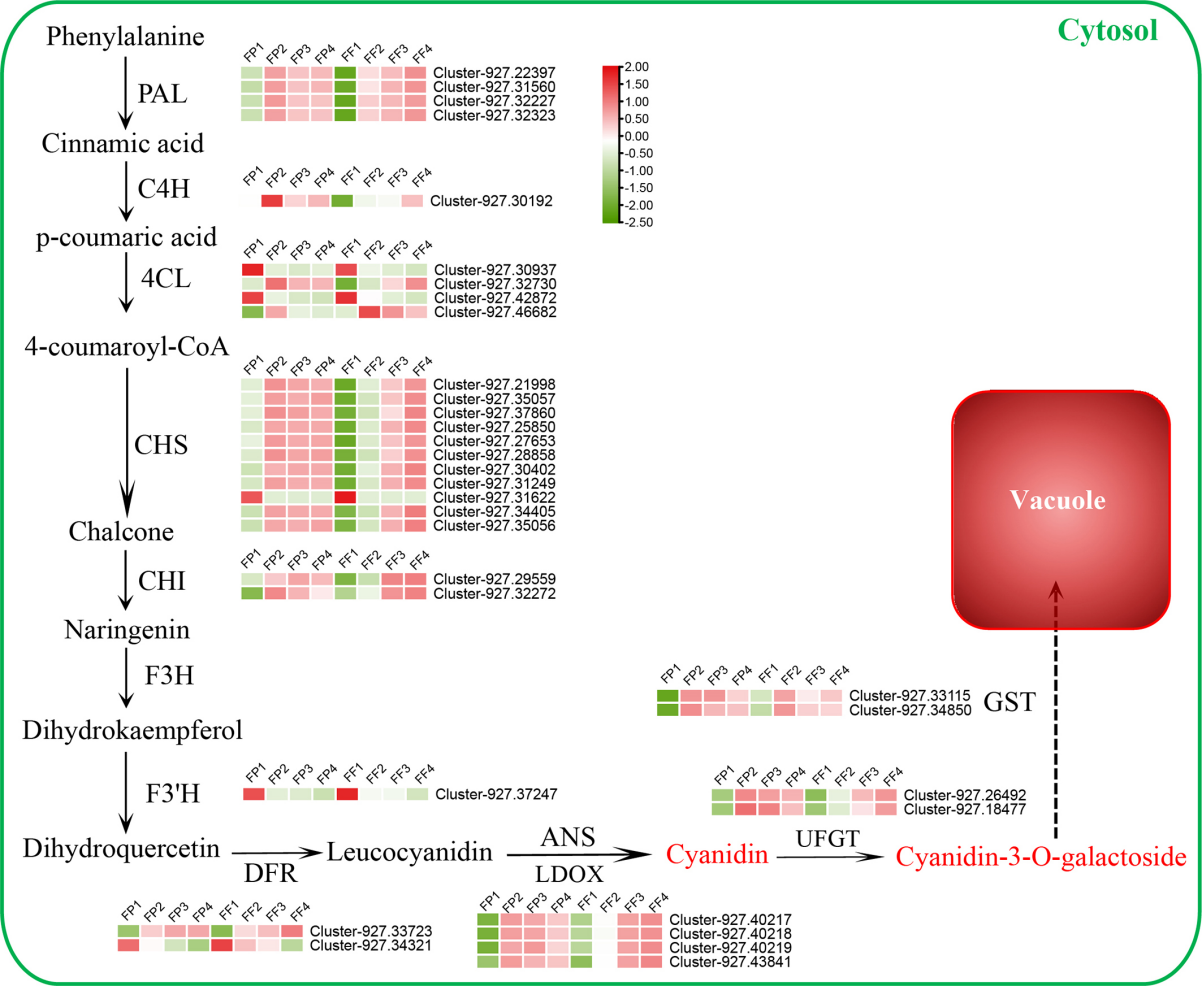
Differentially expressed structural genes regulatory network of cyanidin biosynthesis. The expression level from low to high is represented by green to red.

To explore the biosynthesis pathway of cyanidin during the post-harvest color conversion of the ‘Jinhongguan’ fruit, we conducted a screening of the genes related to anthocyanin biosynthesis in 5751 common DEGs in the transcriptome date. A total of 109 anthocyanin biosynthesis-related genes were identified, including 36 structural genes and 73 MBW TFs (**Table S6**). Among the 36 structural genes, 33 were associated with cyanidin biosynthesis, and the expression heatmap of these genes was carried out. (**Figure 5**). Among them, 19 structural DEGs related to the early enzyme reaction were found to be significantly up-regulated, including four *PAL* genes, one *C4H* gene, two *4CL* genes, ten *CHS* genes and two *CHI* genes (**Figure 5**; **Table S6**). Most of these structural genes exhibited low expression levels in stage 1 of both peel and flesh, and their expression levels increased significantly in subsequent stages. In addition, four early cyanidin biosynthesis-related genes were down-regulated, including two *4CL* genes (Cluster-927.30937, Cluster-927.42872), one *CHS* gene (Cluster-927.31622) and one *F3’H* gene (Cluster-927.37247), which had the highest expression levels in stage 1 of both peel and flesh. Among the late biosynthetic genes of cyanidin, one *DFR* gene, four *LODX* genes, two *UFGT* genes and two *GST* genes were significantly up-regulated in stage 2/3/4 of both peel and flesh. *DFR* is a key enzyme that catalyzes the synthesis of colorless leucocyanidin from dihydro quercetin (Tanaka et al., 2008). In this study, *AaDFR1* (Cluster-927.33723) was significantly up-regulated in stage 2/3/4 of both peel and flesh. Leucocyanidin is oxidized and dehydrated under the catalysis of *LODX*/*ANS* to form a colored unstable cyanidin (Jaakola, 2013). Here, four *LODX* genes (Cluster-927.40217, Cluster-927.40218, Cluster-927.40219 and Cluster-927.43841) were found to be highly induced in stage 2/3/4 of peel and flesh. These *LODX* genes are likely responsible for catalyzing the conversion of colorless anthocyanins into cyanidins. Subsequently, the unstable cyanidin requires UFGT to catalyze its glycosylation to become stable (Jaakola, 2013). Our data showed that two *UFGT* genes (Cluster-927.26492 and Cluster-927.18477) were induced during fruit color conversion, of which Cluster-927.26492 encodes a 3-O-beta-D-galactosyltransferase, and Cluster-927.18477 encodes a 3-O-beta-D-xylose galactosyltransferase. Metabolomics data indicated that cyanidin-3-O-galactoside was the main pigment responsible for the red color of the flesh in ‘Jinhong guan’. Based on these findings, we speculated that cyanidin might be mainly catalyzed by Cluster-927.26492 to stable cyanidin-3-O-galactose. In addition, two *GST* genes (Cluster-927.33115 and Cluster-927.34850) were significantly induced during the peel and flesh color conversion process, suggesting their involvement in the transport of cyanidin-3-O-galactose to vacuoles. The above results indicated that these 33 structural genes might be essential for the biosynthesis of cyanidin in ‘Jinhongguan’ fruit and participate in the process of fruit color conversion.

### 3.6 Analysis of differentially expressed transcription factors related to cyanidin synthesis pathway

**Figure 6:**
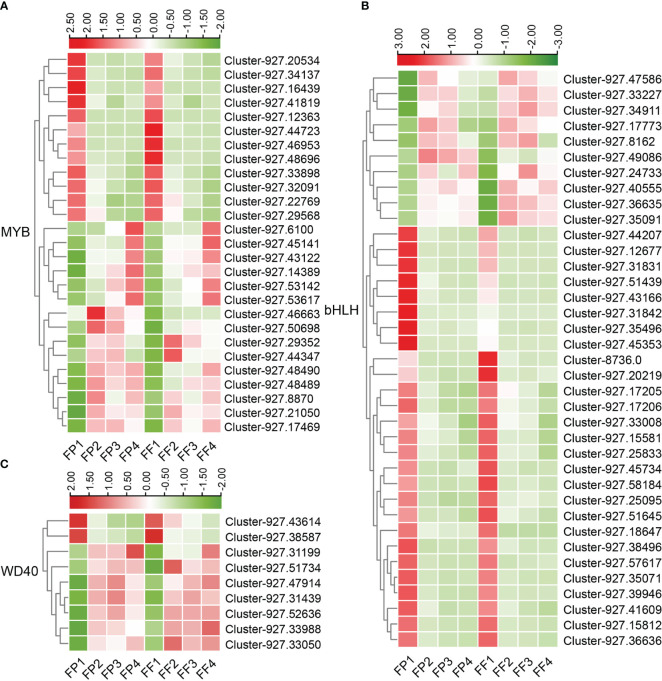
Heatmap of differentially expressed MBW transcription factors in the transcriptome. **(A-C)** Heatmap of 27 differentially expressed MYB TFs **(A)**, 37 differentially expressed bHLH TFs **(B)** and 9 differentially expressed WDR TFs **(C)**.

The biosynthesis of anthocyanins is regulated by various TFs, the most important of which are MYB, bHLH and WDR. In this study, a significant number of the 5751 DEGs were TFs, and 27 MYB TFs, 37 bHLH TFs and 9 WDR TFs were identified (**Figure S1**). The expression heatmap revealed that most of the MYB and WDR TFs were up-regulated (15 of MYB TFs, 7 of WDR TFs), while most of the bHLH TFs (27 out of 37) were down-regulated (**Figure 6**; **Figure S1**). Some TFs, such as Cluster-927.46953 (MYB), Cluster-927.31831 (bHLH), Cluster-927.43614 (WDR), exhibited down-regulated expression patterns in stage 2/3/4, while their expression levels in stage 1 (FP1 and FF1) were significantly higher (**Figure 6**), indicating a negative correlation with anthocyanin biosynthesis. Moreover, the expression peaks of the up-regulated TFs appeared in different stages. For example, Cluster-927.21050 (MYB) and Cluster-927.47586 (bHLH) reached their expression peak in stage 2 (FP2 and FF2), while Cluster-927.34911 (bHLH) and Cluster-927.31439 (WDR) peaked in stage3 (FP3 and FF3), Cluster-927.6100 (MYB) and Cluster-927.31199 (WDR) peaked in stage 4 (FP4 and FF4) (**Figure 6**). These results implied that these TFs might promote the biosynthesis of cyanidin by regulating the expression of structural genes at different stages.

### 3.7 Correlation analysis between the transcriptome and metabolome data

**Figure 7:**
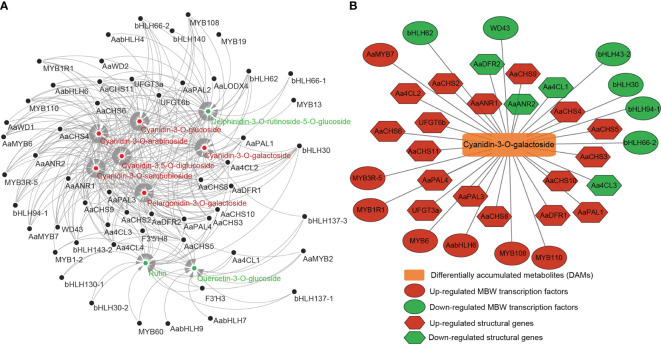
The correlation between differentially accumulated metabolites and differentially expressed structural genes and MBW transcription factors. **(A)** The correlation between 9 DAMs and 26 structural DEGs and 29 MBW TFs. Red: up-regulated DAMs, green: down-regulated DAMs. **(B)** The correlation between cyanidin-3-O-galactoside and 21 structural DEGs and 13 MBW TFs.

To further clarify the relationship between DEGs and DAMs, the correlation between nine anthocyanin DAMs and 36 anthocyanin synthesis structure DEGs or 73 MBW TFs was analyzed. The results showed that nine DAMs were significantly correlated with 26 structural DEGs and 29 MBW TFs. There were 266 significantly correlated pairs, of which 90 pairs were negatively correlated, and 176 pairs were positively correlated (**Figure 7A**; **Table S7**). Some DEGs were significantly correlated with 8 DAMs simultaneously, such as *AaPAL3*/*4*, *Aa4CL2*/*4*, *AaCHS2*/*3*/*5*/*9*/*10*, *AaDFR2* and *bHLH30* (**Figure 7A**; **Table S7**). Except for *bHLH30*, all these genes belonged to early structural genes involved in anthocyanin biosynthesis, highlighting the crucial role of early structural genes in anthocyanin biosynthesis. Furthermore, seven out of the nine DAMs showed significant correlations with more than 30 DEGs, while the remaining two down-DAMs were correlated with 14 and 23 DEGs, respectively (**Table S7**). Given that cyanidin-3-O-galactoside was the most abundant pigment in the fruit color conversion of ‘Jinhongguan’ (**Figure 4B**), the correlation between cyanidin-3-O-galactoside and structural DEGs and MBW TFs was analyzed. A total of 34 DEGs (21 structural DEGs and 13 MBW TFs) were significantly correlated with cyanidin-3-O-galactoside, most of which were positively correlated (**Table S7**). Ten DEGs showed negative correlation with cyanidin-3-O-galactoside, half of which belonged to the bHLH TF family. Only *AabHLH6* exhibited a positive correlation with cyanidin-3-O-galactoside, while all six MYB TFs were positively correlated with it (**Figure 7B**). These findings suggested that bHLH TFs might mainly negatively regulate cyanidin-3-O-galactoside synthesis, while MYB TFs might positively regulate it.

### 3.8 Quantitative analysis of differentially expressed structural genes and MBW transcription factors related to cyanidin biosynthesis

**Figure 8:**
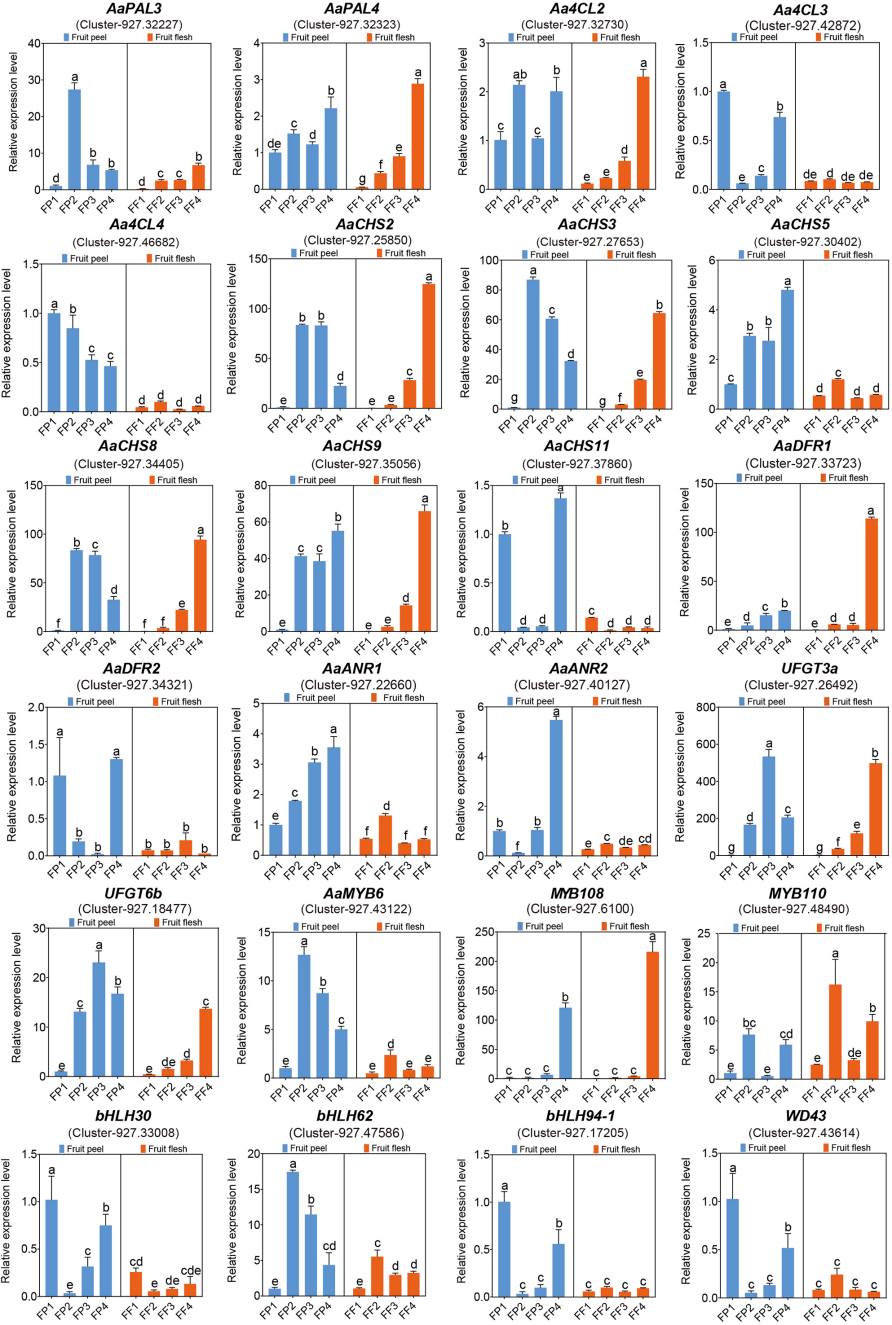
The qRT-PCR analysis of candidate genes related to anthocyanin biosynthesis. The significantly different expression levels of genes in fruit peel and flesh among different stages were evaluated by One-way ANOVA and Duncan test (p < 0.05). Bars with the same lowercase letters indicate no significant difference, while bars without shared lowercase letters indicate significant difference.

To further screen structural genes and MBW TFs, we performed qRT-PCR analysis of 24 genes based on transcriptome and metabolome data. Among the 17 structural genes, *AaPAL3*, *AaCHS2*/*3*/*8*/*9*, *UFGT3a* and *UFGT6b* were significantly highly expressed in peel and flesh in stage 2/3/4, reaching their peak in flesh at FF4 (**Figure 8**), which was consistent with the phenotype of the flesh completely turning red at FF4 (**Figure 1B**). This indicated that these seven structural genes were closely related to the biosynthesis of cyanidin in ‘Jinhongguan’. The expression level of *AaANR1* gradually increased from FP1 to FP4 with higher expression in the peel than in the flesh. Its highest expression was observed in FP4, while *AaDFR1* exhibited the highest expression in FF4 (**Figure 8**), indicating that the structural genes involved in the synthesis of cyanidin might differ between the peel and flesh. Regarding the MBW TFs, the expression patterns of *bHLH30*, *bHLH94-1* and *WD43* were similar to those of structural genes *Aa4CL3*, *AaCHS11* and *AaDFR2* (**Figure 8**), indicating that these TFs might directly bind to the promoters of these structural genes to regulate their transcription, thus promoting cyanidin biosynthesis. *MYB108* showed significantly higher expression in FP4 and FF4 compared to other stages. (**Figure 8**), suggesting its potential role in the transport and accumulation of cyanidin. Taken together, all the above results indicated that structural genes including *AaPAL3*, *Aa4CL3*, *AaCHS2*/*3*/*8*/*9*/*11*, *AaDFR1*/*2*, *AaANR1*, *UFGT3a* and *UFGT6b*, as well as TFs including *MYB108*, *bHLH30*, *bHLH94-1* and *WD43* played important roles in cyanidin biosynthesis in ‘Jinhongguan’.

## 4 Discussion

Kiwifruit flesh is rich in color, exhibiting extensive variations among different species and within species. Currently, the cultivated varieties of kiwifruit mainly consist of *Actinidia deliciosa* with green flesh (such as ‘Hayward’), *Actinidia chinensis* with yellow flesh (such as ‘Hort16A’) or red flesh core (such as ‘Hongyang’, ‘Donghong’), and *Actinidia arguta* with red flesh (such as ‘Hongbaoshixing’) (McGhie and Ainge, 2002; Nishiyama, 2007). The main pigments in the green, yellow and red flesh of kiwifruit are chlorophyll, carotene and anthocyanin, respectively, and pigment content, proportion and spatial distribution determine the color of kiwifruit (Nishiyama, 2007; Montefiori et al., 2009). Initially, the flesh color of yellow and green kiwifruit is green at the early stage of development. As the fruit matures, the chlorophyll in yellow kiwifruit degrades, while its original carotenoid is retained, resulting in a yellow flesh color. By contrast, the content of chlorophyll in green kiwifruit remains unchanged, maintaining a green flesh color throughout ripening (Pilkington et al., 2012). Most kiwifruit cultivars have completed the color conversion process before reaching maturity. However, in this study, the fruit peel and flesh of ‘Jinhongguan’ (a new cultivar of *Actinidia arguta*) gradually turned fully red during storage in the dark at 25°C for about one week, which differs from previous studies. Anthocyanin was identified as the main pigment in the ‘Jinhongguan’ fruit, and total anthocyanin content was higher in the peel than in the flesh at the same storage stage, which may be due to the higher water content in the flesh than that in the peel. Previous studies have identified cyanidin-based anthocyanins as the pigments in red-fleshed kiwifruit (Montefiori et al., 2011). Recent studies have also reported the presence of a small number of delphinidin derivatives in the flesh of *Actinidia melanandra* and *Actinidia arguta*, as well as in the peel of *Actinidia chinensis* ‘Hongyang’ (Montefiori et al., 2009; Liu et al., 2017). Similarly, in this study, the anthocyanin-targeted metabolome analysis revealed that the main pigment in the flesh of ‘Jinhongguan’ was cyanidin, and a small number of pelargonidins were also detected. In addition, cyanidin mainly exist in the form of cyanidin-3-O-galactoside, and pelargonidin exist as pelargonidin-3-O-galactoside. These findings have further revealed the composition and existence form of pigments in red-fleshed kiwifruit.

The genes related to anthocyanin synthesis mainly include the structural genes encoding various enzymes and the TFs regulating the structural genes (Liu et al., 2016; Wang et al., 2022b). Some structural genes and TFs have been confirmed to be involved in the synthesis of anthocyanin in kiwifruit. For example, in *Actinidia arguta Sieb. Zucc*, *AaF3H*, *AaLDOX*, *AaUFGT*, *AaMYB*, *AabHLH* and *AaHB2* have been reported to regulate flavonoid biosynthesis in ‘Hongbaoshixing’ and *AaLDOX* controls anthocyanin biosynthesis in the flesh of ‘Tianyuanhong’ (Li et al., 2018a; Li et al., 2018b). The cMYBF110-AcbHLH1-AcWDR1 complex can bind to the promoters of structural genes including *AcCHS*, *AcF3′H*, *AcANS*, *AcUFGT3a*, *AcUFGT6b* and *AcGST1* to activate their expression (Liu et al., 2021). In this study, transcriptome analysis of ‘Jinhongguan’ identified a total of 5751 common DEGs in the peel and flesh. Furthermore, 36 structural DEGs and 73 MBW TFs related to anthocyanin synthesis were screened by gene annotation, KEGG and GO enrichment analysis. The expression heatmap analysis showed that multiple structural genes related to the catalyzed reactions of anthocyanin biosynthesis were significantly up-regulated, such as *PAL*, *C4H*, *4CL*, *CHS*, *CHI*, *DFR*, *LDOX*, *UFGT* and *GST* genes. These genes exhibited low expression levels in stage 1 of peel and flesh and were increased significantly increased in the late stages, indicating that these structural genes were functionally conserved and were closely related to anthocyanin synthesis in ‘Jinhongguan’. Among the 5751 common DEGs, 27 MYB TFs, 37 bHLH TFs, and 9 WDR TFs were identified. The expression heatmap also demonstrated that most of the MYB and WDR TFs were up-regulated, while the majority of bHLH TFs were down-regulated. These down-regulated TFs might have a negative regulatory role in anthocyanin synthesis. At present, some TFs have been reported to negatively regulate anthocyanin synthesis. For example, *PtrMYB57* and LcbHLH92 are negative regulators of anthocyanin/proanthocyanidin accumulation in Poplar and Sheepgrass (*Leymus chinensis*), respectively (Wan et al., 2017; Zhao et al., 2018). In *Chimonanthus praecox* (L.), ectopic overexpression experiments in model plants have revealed that *CpbHLH1* can reduce anthocyanin accumulation by inhibiting the expression of genes involved in the flavonoid biosynthesis pathway (Zhao et al., 2020).

To further screen cyanidin biosynthesis-related candidate genes, we performed a correlation analysis between the transcriptome and metabolome. A total of 21 structural DEGs were significantly correlated with cyanidin-3-O-galactoside metabolism, most of which exhibited a positive correlation. Notably, *AaPAL4*, *Aa4CL2*, *AaDFR2* and *AaCHS2*/*3*/*5*/*9*/*10* were significantly correlated with eight DAMs including cyanidin-3-O-galactoside. These findings suggested that these structural genes were key genes involved in the anthocyanin synthesis process in ‘Jinhongguan’. In addition, 13 MBW TFs including six MYB, six bHLH and one WDR were also significantly correlated with cyanidin-3-O-galactoside. Specifically, all the MYB TFs showed a positive correlation with cyanidin-3-O-galactoside, while five bHLH TFs and one WDR TF showed a negative correlation with this metabolite, indicating that the MYB TFs positively regulated the biosynthesis of cyanidin-3-O-galactoside, while bHLH and WDR TFs mainly negatively regulated it. The qRT-PCR results further confirmed that these structural genes and MBW TFs played important roles in cyanidin biosynthesis in ‘Jinhongguan’. However, the specific functions and regulatory mechanisms of these genes remain to be further explored.

## 5 Conclusion

Here, we used transcriptome and metabolome analysis of anthocyanin biosynthesis in post-harvest fruits of a new full red-type kiwifruit (*Actinidia arguta*) ‘Jinhongguan’. The determination of total anthocyanin content showed that anthocyanin was the main pigment accumulated during the reddening process of the ‘Jinhongguan’ fruit. Additionally, the anthocyanin-targeted metabolome analysis revealed that the main form of anthocyanin was cyanidin-3-O-galactoside. Through correlation analysis between the transcriptome, metabolome and qRT-PCR, 12 structural genes and 4 MBW TFs were identified as key candidate genes responsible for cyanidin-3-O-galactoside biosynthesis. This study provides insight into the composition of anthocyanins in *Actinidia arguta* and lays a foundation for revealing the molecular mechanism of anthocyanin synthesis in post-harvest fruits of *Actinidia arguta*.

## Data availability statement

The original contributions presented in the study are included in the article/**Supplementary Material**, further inquiries can be directed to the corresponding authors.

## Author contributions

LY: Investigation, Funding acquisition, Writing – original draft. FB: Validation, Writing – review and editing, Data curation. LZ: Funding acquisition, Writing – review and editing, Resources. ML: Investigation, Supervision, Writing – review and editing. LG: Investigation, Methodology, Writing – review and editing. ZW: Data curation, Validation, Writing – review and editing. JP: Investigation, Writing – review and editing. QC: Conceptualization, Writing – review and editing. XL: Conceptualization, Writing – review and editing.

## Supplementary material

Supplementary Figure 1 Transcription factor classification statistics of common differentially expressed genes in the transcriptome.
