# Supplementary figures and images for: Transcriptome and metabolome analyses of anthocyanin biosynthesis in post-harvest fruits of a full red-type kiwifruit (Actinidia arguta) ‘Jinhongguan’
Source: Front Plant Sci. 2023 Oct 9;14:1280970. doi: 10.3389/fpls.2023.1280970 (PMC10591155; doi:10.3389/fpls.2023.1280970)

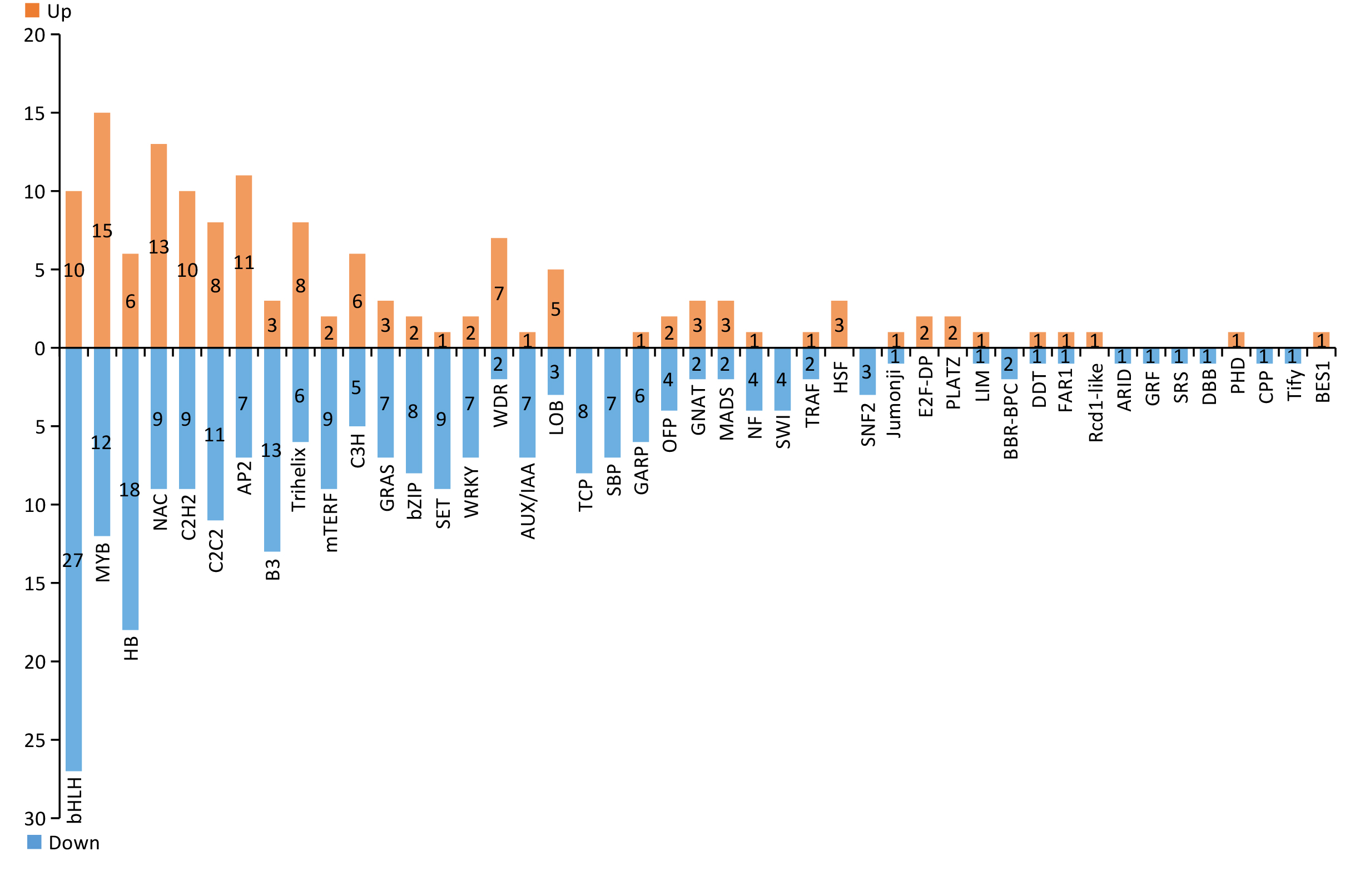

Supplement: Supplementary Figure 1 — Transcription factor classification statistics of common differentially expressed genes in the transcriptome. [file Image_1.jpeg]
